# Candidal speciation and carriage in oral cavity of HIV-positive children/adults and healthy individuals in the South Canara district of India: a comparative study

**DOI:** 10.1186/s13104-025-07190-7

**Published:** 2025-03-27

**Authors:** Sneha KS, Srikant Natarajan, Karen Boaz, Shrikala Baliga, John Ramapuram, Monica Charlotte Solomon, Nidhi Manaktala, Nunna Sai Chitra

**Affiliations:** 1 Department of Oral Pathology and Microbiology, Manipal College of Dental Sciences Mangalore, Manipal Academy of Higher Education, Manipal, Karnataka India; 2 Department of Microbiology, Kasturba Medical College Mangalore, Manipal Academy of Higher Education, Manipal, Karnataka India; 3 Department of Internal Medicine, Kasturba Medical College Mangalore, Manipal Academy of Higher Education, Manipal, Karnataka India; 4 Department of Oral Pathology and Microbiology, Manipal College of Dental Sciences Manipal, Manipal Academy of Higher Education, Manipal, Karnataka India

**Keywords:** HIV, *Candida*, Child, Adults, Biological evolution

## Abstract

**Objective:**

Candidiasis, an opportunistic infection that is prevalent in HIV-positive children and adults, is caused by various *Candida* species, *Candida albicans* along with certain non-albicans *Candida* species. The prevalence of these species varies across age groups due to multiple factors. Identification of these species becomes necessary as any antifungal therapy requires species-specific targeting to manage candidiasis effectively. With this background, the present study aimed to evaluate the prevalence and speciation of *Candida* in HIV-positive children (*n* = 30) and adults (*n* = 40) and to compare these findings with those in healthy individuals. Ten mL saliva was collected from HIV-positive and HIV-negative (healthy) patients and cultured on CHROMAgar™.

**Results:**

The proportion and quantity of candidal colonization was higher in HIV-positive children (93.30%) as compared to adults (67.50%). An increased profile of *Nakaseomyces glabrata (*previously *Candida glabrata)* was seen in children while the adults showed increase in colonization of *C. tropicalis.* The shift in profiles of species from *Candida albicans* to ‘*non-albicans’ Candida* species is of clinical relevance as it directly impacts on the antimicrobial efficacy of chosen anti-fungal agents.

## Introduction

Acquired Immunodeficiency Syndrome (AIDS), caused by Human Immunodeficiency Virus (HIV), is a significant global health issue, with low-income countries spending an average of $70.3 billion on healthcare [1]. The progression of HIV infection is marked by a steady decline in immune function and emergence of opportunistic diseases, including fungal infections, notably, infections caused by *C. albicans*. While the high prevalence of *C. albicans* induced lesions is largely attributed to the immunosuppressive effects of HIV, research suggests that *C. albicans* strains isolated from the oral cavities of HIV-infected individuals exhibit greater virulence compared to those from healthy individuals [2, 3]. Coleman et al., reviewed progress made in the identification and diagnosis of *C. albicans* and the *non-albicans Candida* species in HIV-infected individuals, and reported that *Candida* is rather difficult to characterize and behave in a different way compared to their counterparts in non-HIV individuals [4]. Oral candidiasis, an early sign of HIV, is a key indicator of AIDS, with superficial tissue invasion making it a significant risk predictor [5, 6]. 

Several studies in India have examined the prevalence of candidal species in HIV-infected adults. Kirthi YK (2019) found 20% prevalence of oral candidiasis in Madhya Pradesh [7], while Deb T et al. (2003) reported 22.8% in Manipur [8]. Prasad et al. reported 39% in Mangalore city [9], Maheshwari et al. (2016) identified 128 candidal isolates from 88 cases, revealing 7 species: *C. albicans* (50%), *Nakaseomyces glabrata (N. glabrata)* (17%), *C. dubliniensis* (12.5%), *C. parapsilosis* (7.8%), *Pichia kudriavzevii*, (*P*. *kudriavzevii)*,* C. tropicalis* (4.6% each), and *Kluyveromyces marxianus* (3%) [10]. Jayachandran et al. (2017) showed *C. albicans* was the most common species in 56.45% of cases, followed by *C. tropicalis* in 19.3% [11]. 

Majority of the studies focus on HIV- infected adults. Research on candidiasis in HIV-infected children remains limited. It is known that children and adults have different immunological profiles; the immune system in children is nascent and develops with age. Infection with HIV due to a vertical transmission from the mother has more drastic implications for an infected child as compared to HIV-infected adults [12]. 

Research has shown an increase not only in *Candida albicans* but also in rare species such as *Wickerhamomyces anomalus*, *P. kudriavzevii*, and *C. infanticola*,* C. spencermartinsiae* etc [13]. The recurrent episodes of opportunistic infections (OI) necessitate repeated use of antifungal and antimicrobial drugs, leading to the emergence of these *non*-*albicans Candida* species in HIV-positive individuals. The *non*-*albicans Candida* species require distinct treatment approaches, as they develop resistance to antifungal drugs through various mechanisms.

Therefore, this study was conducted with the hypothesis that colonization patterns would differ between children and adults. We aimed to assess the prevalence of various *Candida* species through culture and identify distinct species in HIV-positive children and adults, while comparing their prevalence with that in healthy individuals. Speciation of *Candida* in HIV-positive individuals is crucial for identifying specific pathogenic strains, guiding targeted antifungal therapy, and monitoring the risk of opportunistic infections and antifungal resistance. Comparing *Candida* species in HIV-positive children and adults is significant for understanding age-related differences in colonization patterns, immune responses, and susceptibility to opportunistic infections, which can inform tailored treatment strategies and improve patient outcomes.

## Materials & methods

### Study cohort

This cross-sectional study included 30 cases of HIV-positive children (aged 8–16 years) from a care home for HIV-positive children at Mangaluru, Karnataka, India. In this time-bound study, 40 adults (age *≥* 30 years) were examined from the outpatient and inpatient visits at our Medical College and Hospital. The study included confirmed cases of HIV-positive adults and children who were on anti-retroviral therapy (ART), while HIV-positive patients using antifungal agents or mouthwashes were excluded. The study cohort also excluded patients with any dental prosthesis/dental appliances that would interfere with the expression of *Candida* species. Age and sex-matched controls were taken for children and adults for the first 10 samples. Healthy children (4–12 years) and healthy adults (24–42 years) from the same geographical location and negative for HIV-infection were taken as control samples. Demographic data, CD4 counts, history of opportunistic infections, medications taken by the HIV infected participants were retrieved from the case sheets of the participants.

### Ethical approval and informed consent

All subjects in the study were **informed** regarding the procedure and **written consent** was taken from every patient. In case of children, permission and **written informed consent** were obtained from legally authorized representative (LAR) of the care home prior to commencement of the study. For participants aged 16 years and older, the objectives of the research and the method of its implementation were fully **informed** to the eligible people, and **written consent** was obtained prior to sample collection. The study was approved by Institutional Ethics Committee, Manipal College of Dental Sciences Mangalore (ref no:15103) and conformed with the principles of the Helsinki Declaration.

### Sample collection

Each patient was given a sterile rubber band to chew for a minute to stimulate saliva production. Patients were asked to expel the stimulated saliva in a sterile bottle from which 10 mL of saliva was taken for culture to the Microbiology Laboratory within half an hour of collection.

### Culturing micro-organisms

**Fig. 1:**
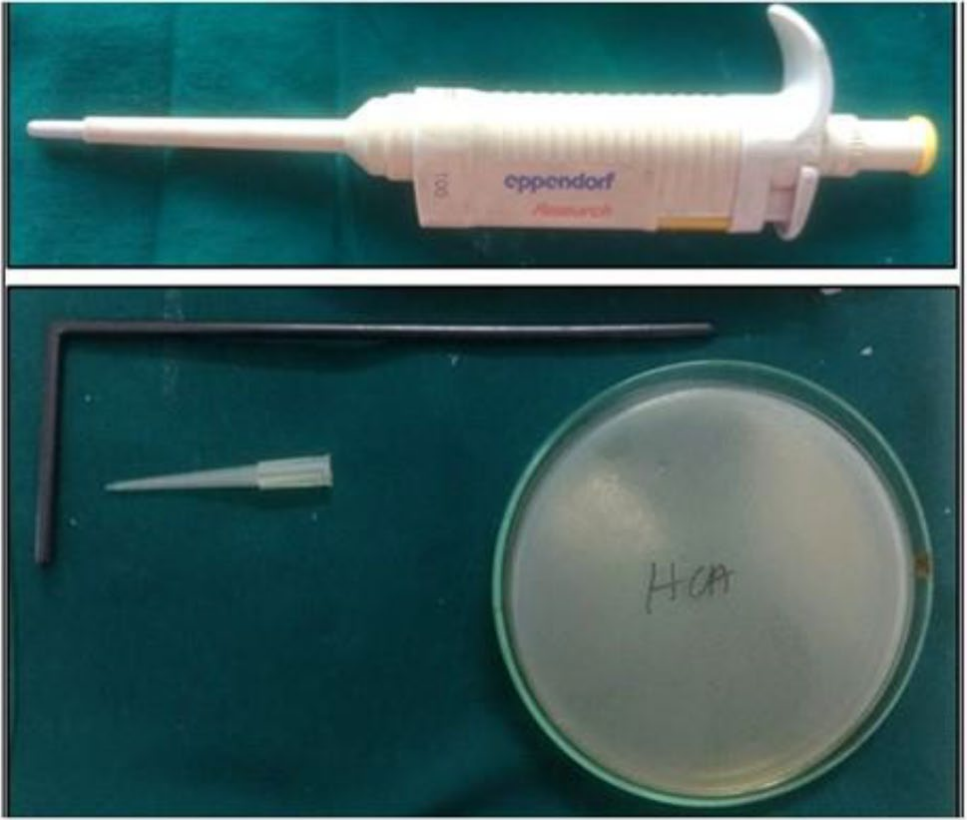
Armamentarium for fungal microbial culture: micropipette, L-shaped cell microbiological spreaders for spreading samples on agar surfaces in Petri dishes, and high CHROMAgar

Culturing of *Candida* species was done at Microbiology department, Kasturba Medical College and Hospital, Mangalore. Culture plates (in triplicate), were prepared 2 days prior to the sample collection, coded with the name, age and gender of the patients and controls. Of the 10 mL saliva collected, 100 µL was taken with a micropipette (Fig. 1) and spread onto CHROMAgar^™^ plates directly in four directions with the help of a sterile plastic spreader. No other culture medium was used prior to this.

**Fig. 2:**
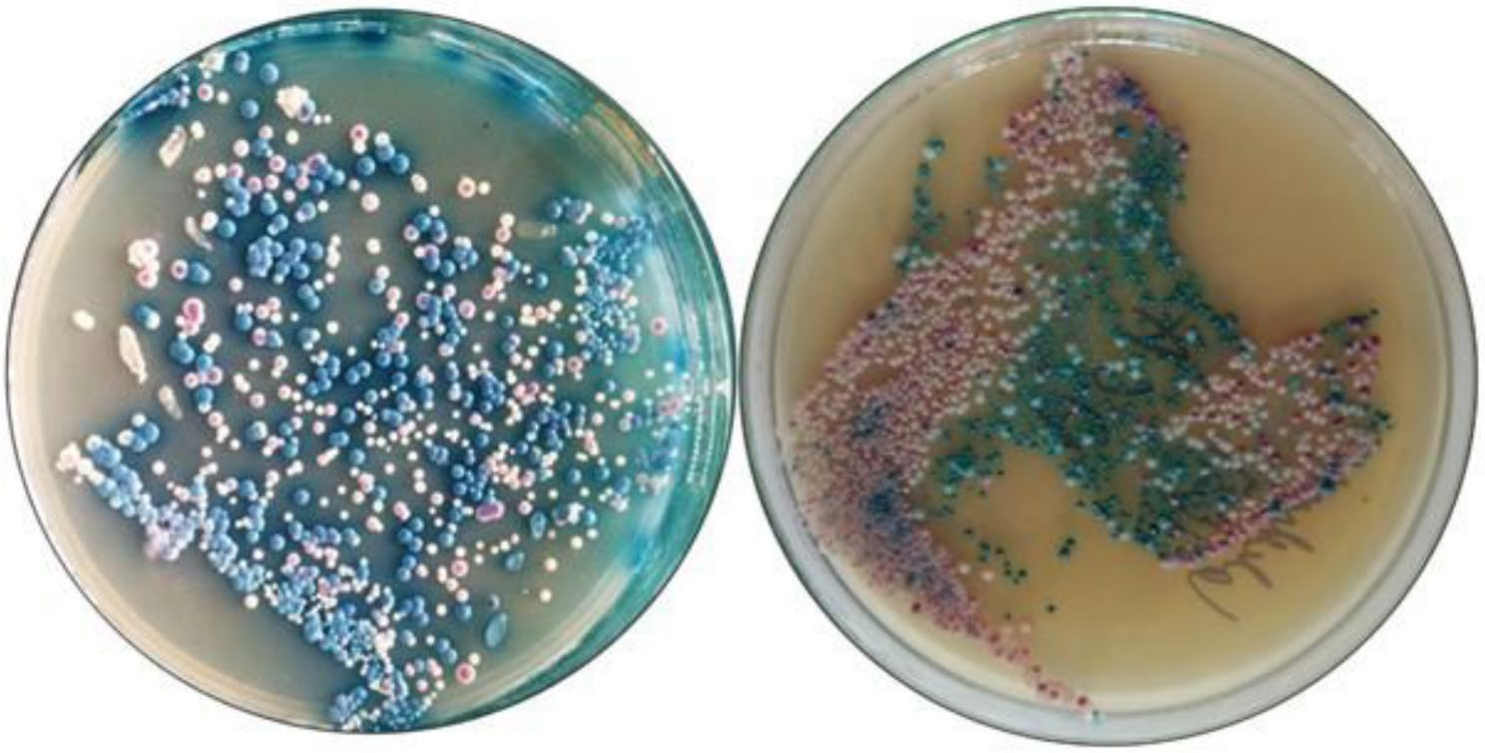
Collected samples showing a mixture of species as seen in CHROMagarTM *C. albicans* (smooth light green-colored colonies),. *N. glabrata* (cream to white smooth colonies); *C. tropicalis* (metallic blue colored raised colonies) and *P. kudriavzevii* (purple fuzzy colonies)

The plates were then transferred to an incubator where the colonies were allowed to grow at room temperature (24–32° Celsius) for 48 h. Multiple species of *Candida* were differentiated by the color of the colonies on CHROMAgar^™^[Catalogue number CA223-25 25 L pack and manufactured by 4 place du 18 juin 1940 75006 Paris, www.CHROMagar.com]. Co-existence of the various species was analyzed from the culture in the same plate. While *C.albicans* exhibited light green-colored smooth colonies, *C.tropicalis* showed blue to metallic blue-colored raised colonies; *N.glabrata* had cream to smooth white colonies while *P.kudriavzevii* showed purple ill-defined colonies. (Fig. 2) The speciation was confirmed using VITEK ^®^2 COMPACT system [3, 14]. Species identified in 100 µL of saliva were counted, multiplied by a factor of 10 to get the number of colonies in 1 mL of saliva to express the colonies in terms of colony forming units per mL (CFU/mL).

### Statistical analysis

The collated data was statistically evaluated using SPSS 20. Proportion of patients showing positive colonization in terms of various *Candida* species and in total between child/adult cases and controls were compared using chi-square tests. Due to skewed distribution of number of colonies between adults and children, the median number of colonies between controls and cases as well as the adult and child HIV-infected individuals were compared using non-parametric Mann-Whitney U test. The *Candida* colonization was compared between the four categories of CD4 counts using Kruskal-Wallis test.

## Results

Among the 70 HIV-positive participants, 30 were children (8–16 years), with 43% females and 56% males. Most adult participants were classified as WHO clinical stage I (77.5%). Analysis of CD4 counts showed that 53.3% of the children had a CD4 count above 750 cells/cu.mm, whereas majority of adults (40%) had CD4 counts ranging from 251 to 500 cells/cu.mm. (Table 1)

**Table 1:**
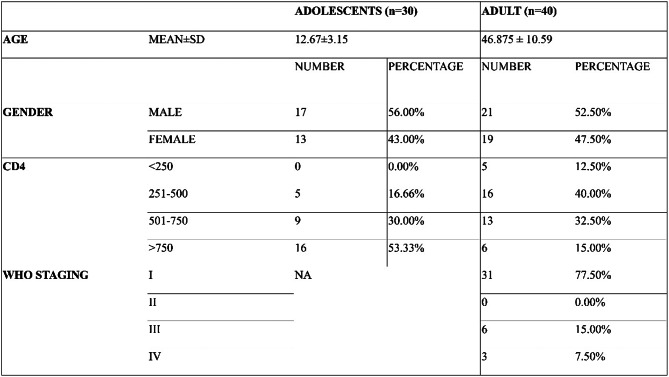
Demographic data of the patient cohort

*Candida* prevalence was significantly higher in HIV-positive participants compared to healthy controls. In children, 93.3% of HIV-positive cases had *Candida* colonization, versus 30% in controls (*p* = 0.000033), and in adults, 67.5% of HIV-positive cases showed colonization, compared to 30% in controls (*p* = 0.03). *C. albicans* and *N. glabrata* were notably more common in HIV-positive children, at 53.3% and 66.7% (*p* = 0.003 and < 0.001). Although *P. kudriavzevii* and *C.tropicalis* appeared in both groups, differences were not significant. Among adults, *C.tropicalis* was most frequent in HIV cases (57.5%), while *P. kudriavzevii* and *N.glabrata* were also common but not significantly so (Table 2).

**Table 2:**
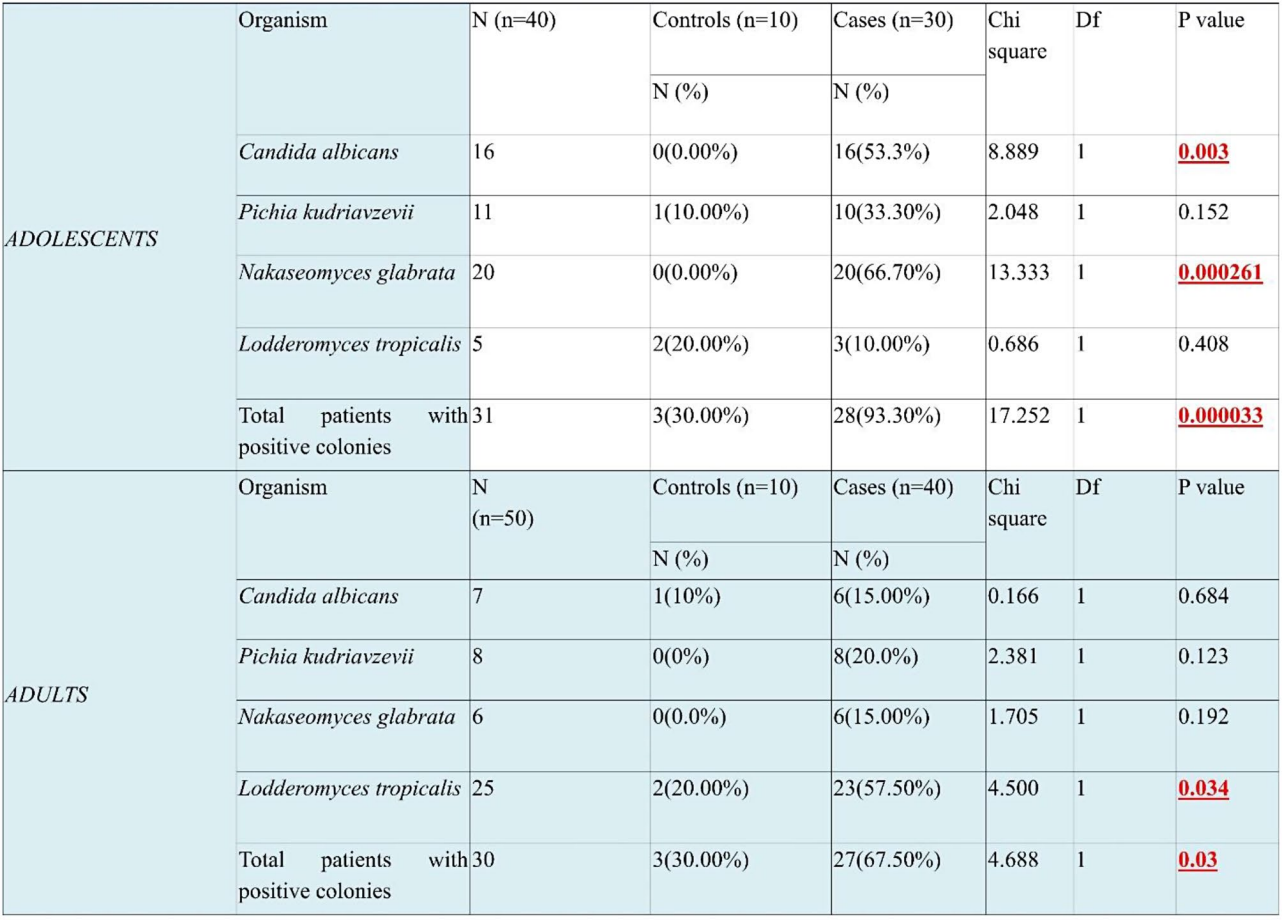
Prevalence of different species of *Candida* adolescents and adults: Chi square test for comparison between cases and controls

Colony-forming units (CFU) were significantly higher in HIV-positive children (median = 165) than in controls (median = 0, *p* < 0.001). *C.albicans* and *N. glabrata* also had higher median counts in cases (10 and 15, respectively, *p* = 0.011 and 0.001). However, *P. kudriavzevii* and *C.tropicalis* showed no significant difference between cases and controls. Among adults, HIV-positive cases had a significantly higher total CFU (median = 50) compared to controls (median = 0, *p* = 0.018). (Table 3)

**Table 3:**
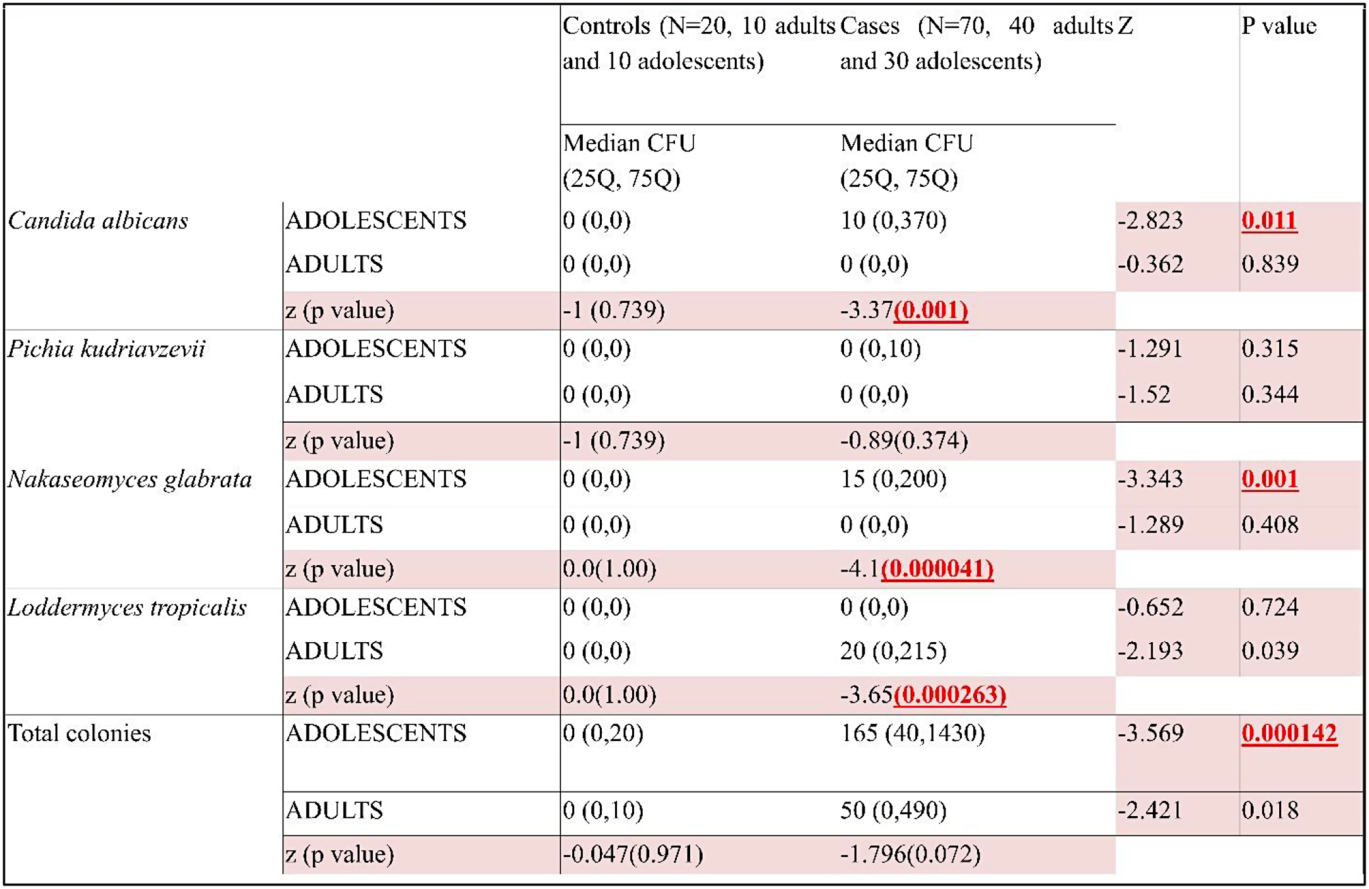
Comparison of candidal carriage in CFU between cases and controls of adolescents/adults and between HIV positive adolescents and adults using Mann Whitney U test

In adults, *C.tropicalis* was significantly higher in cases (median = 20) than controls (median = 0, *p* = 0.039). *C.albicans*, *P.kudriavzevii*, and *N.glabrata* showed no significant difference between adult cases and controls. Total *Candida* colonies were higher in children (median = 165) than adults (median = 50), with *C. albicans* and *N.glabrata* significantly more prevalent in children. *P. kudriavzevii* showed no significant difference between HIV-positive adults and children but was slightly more common in children. (Table 3)

Kruskal-Wallis test revealed no significant difference in *Candida* colonization across the four CD4 categories in both adults and children. The median values in the ‘<250 CD4 category’ were zero, due to a lack of cases in children and very few (*n* = 5) in adults. The total number of colonies was highest in the ‘251–500 CD4’ category for both groups, with counts decreasing as CD4 levels increased. In all categories, children showed higher colonization than adults. Our study found significant differences in Candida species colonization between cases and controls, and between children and adults. In children, 56.7% of HIV-positive cases (17/30) had multiple species colonization, compared to 0% in controls (*p* < 0.001). In adults, 25% of HIV-positive cases (10/40) had multiple species, while none of the controls did (*p* = 0.060). Children were more likely to have multiple species than adults (56.7% vs. 25%, *p* = 0.007), highlighting the higher prevalence of Candida colonization in HIV-positive children. (Table 4)

**Table 4:**
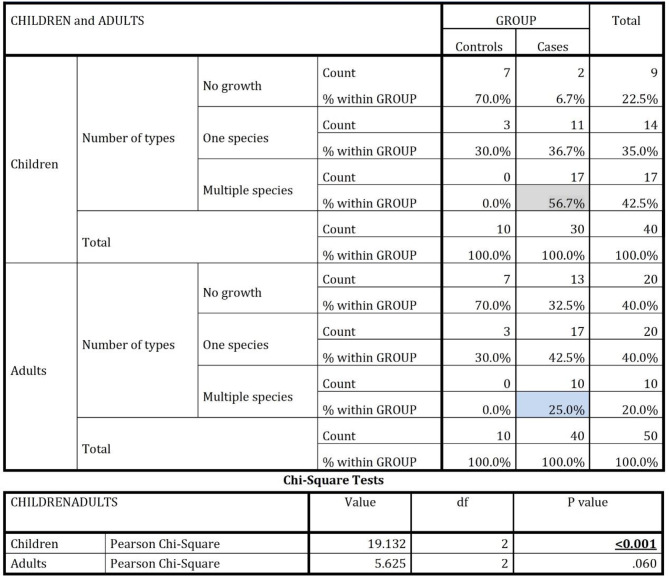
Comparison of distribution of candidal species in HIV positive children and adults using chi-square test

## Discussion

Opportunistic infections are a hallmark of HIV disease. As a precursor to any clinical infection bacterial/fungal organisms are known to grow and colonize the mucosa asymptomatically. In the present study, this asymptomatic colonization (i.e., absence of clinical manifestation of candidiasis) was assessed in patients with comparable levels of immune suppression. The status of immune suppression was established on the basis of CD4 levels which were retrieved from the case sheets of the HIV-positive participants.

In this study, an increasing trend of asymptomatic colonization of *Candida* was observed in HIV-positive children and adults. We observed differences in speciation between the two age groups, which to our notice has never been documented in literature. The study cohort included confirmed cases of HIV-infection in both children and adult population. The study excluded patients with co-morbidities, patients on anti-fungal medications, mouthwashes, and patients with dental prosthesis as these factors can cause interference in expression of different *Candida* profiles [15]. 

Established comparison between different age groups confirmed an increase in colonization in HIV-positive children [12]. There exists differences in characteristics of infection between children and adults as compiled by Fidel et al. (2011) and Kukoyi et al. (2016) [16, 17]. Nevertheless, in general the oral microbiome in children remains very different from adults. In young children the oral fungal and bacterial biodiversity is lower and there is less competition when an opportunistic microbe colonizes the oral tract. The present study is novel as no studies have been done till date, comparing adults and children. Furthermore, this is the first study done in the Dakshin Kannada district of the South of India. None of the studies done before, have shown asymptomatic colonization which was seen in our study.

Asymptomatic colonization or absence of clinically evident candidiasis has been one of the important findings in our study. Due to HIV-infection, the cell adhesion molecules (CAMs) of the epithelium including MAdCAM (Mucosal addressin cell adhesion molecule) and E-cadherins are altered. This leads to dysregulation of CD8 + T cell migration (CD8 + T-cells are unable to migrate past lamina propria and thus colonize at basement membrane zone) ultimately causing increased colonization of fungal organisms at surface of the epithelium [17–20]. 

McNulty et al. (2005) compared E-cadherin expression in patients with and without opportunistic candidal infection and found significant reduction in E-cadherin in epithelium of patients showing opportunistic infection [21]. Thus, lower level of epithelial E-cadherin is thought to be a factor in local tissue microenvironment responsible for increased colonization of *Candida*. Goomer, Maris & Amiel (1998) studied age-related expression of cadherin in rabbits and found that young rabbits had decreased cadherin expression as compared to the adult rabbits [22]. This analogy resonates in the present study where increased colonization was seen in children presumably due to lower expression of cadherin.

Studies have also shown the participation of epithelial cells in host defense by production of antimicrobial peptides and pro-inflammatory cytokines. It’s also seen that in infections caused by *C. tropicalis* and *N. glabrata*, the host defenses could be strengthened by the release of perforins and granzymes by polymorphonuclear neutrophils recruited at the site of infection [23]. 

The most frequent organisms seen in HIV-infected individuals belong to the *Candida* species. *Candida* fungus that exists in various forms as normal commensal in humans, manifests itself pathogenic in diseased states or in compromised health. While the predominant species noted in the present study was *C. albicans*, we also found an increasing colonization by *N. glabrata*, *C. tropicalis* and *P. kudriavzevii* in HIV-positive children and adults. Our findings were in conjunction with many studies [10, 23–25]. 

A prevailing theory for increase in *non-albicans Candida* species in HIV-positive patients is the widespread use of antifungals and broad-spectrum antibiotics. Immunosuppression caused by AIDS is often managed with these treatments, primarily targeting *C. albicans*, the predominant species during initial stages of candidiasis. As colonization of *C. albicans* decreases, competitive inhibition at the oral mucosal surface is reduced, allowing for asymptomatic colonization by less virulent *Candida* species like *C. tropicalis*,* N. glabrata*, and *P. kudriavzevii;* collectively referred to as *‘non-albicans’ Candida* species. However, in our study, cases with such treatment regimens were excluded, yet we still observed an increase in *non-albicans Candida* species. This suggests that factors like heightened levels of immunosuppression, and complexity of managing subclinical conditions, may also contribute to emergence of these *non-albicans Candida* species [26]. However, probiotics and antimicrobial photodynamic therapy can be tested in future for treatment [15]. 

Our study used CHROMagar as a culture media for identification of species. Once identified on CHROMagar, confirmation of the same was done by using the VITEK ^®^2 COMPACT system [13, 27, 28] CHROMagar was the primary media in our study as it yields good results in speciation of mixed *Candida* species. The versatility and economy of this media has already been proved by Mathavi et al. (2016) and Eraso et al. (2006) strengthening its efficacy to be used as a screening tool for diagnosing various fungal species as different species have different course of spread, and response to various anti-fungal agents [29, 30]. Thus, being cognizant of species variation in HIV-positive individuals is important, as this alters the treatment strategy in opportunistic infections.

## Limitations

Our study’s age range of 8–16 years in youngsters may have caused variability in the results. The Mangaluru care home provided the sample, therefore we had no control over age selection. Another study limitation was the small sample size of < 250 CD4 count subjects. The patients’ CD4 levels were steady during treatment. This was mostly due to care home patient management. The study found no correlation between CD4 counts and Candida colonization, however this cannot be generalized. The species variance in adults and children is clinically relevant since species manifestation was dynamic even in the small sample size. Since this is an emerging result in our study, a bigger sample size and more research may help explain these discrepancies. Tobacco/tomato agar using API/MALDI-TOF-MS and other molecular approaches could be used to identify species in future investigations. More antifungal susceptibility testing should be done to explore species resistance trends. In our study, care professionals provided oral hygiene measures to care home children. To guarantee adequate nutrition, the hospital dietitian regulated their diet. However, we noted that food and oral hygiene disparities between children in care and community people were not specifically assessed. Varying these elements may affect oral health and Candida species prevalence, which could be a constraint.

Another drawback of this study is the use of Vitek 2 to identify Candida species, especially *C. albicans* and *C. dubliniensis*. The Vitek 2 system may not accurately identify/misidentify these two species due to its low selective capabilities. *C. dubliniensis* shares many phenotypes with *C. albicans* but has different pathogenicity and resistance, which may affect species-level identification in our study. Due to budget constraints, the study could not employ other culture media. Large-scale research could benefit from sophisticated molecular technologies like PCR to accurately identify and differentiate closely related *Candida* species.

## Conclusion

Opportunistic infections are a hallmark of HIV-infection. The disease progresses much faster in children owing to vertical transmission, adding to the fact that children bear a higher viral load and are more vulnerable to recurrent opportunistic infections. An increase in *non-albicans Candida* species was noted in our study. Shift in profiles of species from *C. albicans* to *non-albicans Candida* species is of clinical relevance as it directly impacts the antimicrobial efficacy of chosen anti-fungal agents.

Speciation of *Candida* in HIV positive children and adults is crucial due to varying pathogenicity of different species. While we were able to speciate *Candida*, why certain species of *Candida* were more prevalent, is an area still unexplored. Since this is an emerging finding, a larger sample size and further research could help clarify the underlying mechanisms driving these differences. Differences in speciation in adults and children can be clinically significant as it helps the clinicians narrow down the antifungal therapy. Since adults had specific species (*C. tropicalis)* treatment could be aimed at using narrow spectrum antifungals. Contrastingly, children had multiple species; broad spectrum antifungals become the treatment of choice. Eventually, the significance of identifying specific *Candida* species lies in a more targeted treatment approach potentially reducing dependence on broad spectrum antibiotics. This knowledge is essential for performing accurate antifungal susceptibility tests which are vital in treating opportunistic infections prevalent in these populations. This study being the first of its kind aims to enhance our understanding and approach to these critical infections.

## Data Availability

The data that support the findings of this study are not openly available due to reasons of sensitivity and are available from the corresponding author upon reasonable request. Data are located in controlled access data storage at the Department of Oral Pathology, MCODS Mangalore, Manipal Academy of Higher Education.

## References

[1] Dieleman JL, Haakenstad A, Micah A, Moses M, Abbafati C, Acharya P, et al. Spending on health and HIV/AIDS: domestic health spending and development assistance in 188 countries, 1995–2015. Lancet. 2018;391(10132):1799–829. 29678342 10.1016/S0140-6736(18)30698-6 PMC5946845

[2] Orlandini RK, Bepu DAN, Saraiva M, da CP, Bollela VR, Motta ACF, Lourenço AG. Are Candida albicans isolates from the oral cavity of HIV-infected patients more virulent than from non-HIV-infected patients? Systematic review and meta-analysis. Volume 149. Microbial Pathogenesis: Academic; 2020. 10.1016/j.micpath.2020.104477 32920148

[3] Bailey R, Scott E. Diagnostic Microbiology. eleventh. Mosby, United States of America; 2002. 786–786 p.

[4] Coleman DC, Bennett DE, Sullivan DJ, Gallagher PJ, Henman MC, Shanley DB, et al. Oral Candida in HIV infection and AIDS: new perspectives/new approaches. Crit Rev Microbiol. 1993;19(2):61–82. 8338619 10.3109/10408419309113523

[5] Shokohi T, Aslani N, Ahangarkani F, Meyabadi MF, Hagen F, Meis JF, et al. Candida Infanticola and Candida spencermartinsiae yeasts: possible emerging species in cancer patients. Microb Pathog. 2018;115:353–7. 29292174 10.1016/j.micpath.2017.12.069

[6] Warrier SA, Sathasivasubramanian S. Human immunodeficiency virus induced oral candidiasis. J Pharm Bioallied Sci. 2015;7(6):S812–4. 26538978 10.4103/0975-7406.163577 PMC4606720

[7] Kirti YK. Prevalence of oral candidiasis in Indian HIV Sero-Positive patients with CD4 + Cell count correlation. Indian J Otolaryngol Head Neck Surg. 2019;71(1):124–7. 30906728 10.1007/s12070-018-1342-3 PMC6401036

[8] Deb T, Singh NB, Devi HP, Sanasam JC. Head and neck manifestations of HIV infection: a preliminary study. J Indian Med Assoc. 2003;101(2):93–5. 12841491

[9] Prasad HKC, Bhojwani KM, Shenoy V, Prasad SC. HIV manifestations in otolaryngology. Am J Otolaryngol. 2006;27(3):179–85. 16647982 10.1016/j.amjoto.2005.09.011

[10] Maheshwari M, Kaur R, Chadha S. Candida species prevalence profile in HIV seropositive patients from a major tertiary care hospital in new Delhi, India. J Pathog. 2016;2016:1–8. 10.1155/2016/6204804 PMC4820622 27092278

[11] Jayachandran AL, Katragadda R, Thyagarajan R, Leela K, Shanmugam K, Chelliah A. Oropharyngeal candidiasis among HIV seropositive patients in Chennai, South India: an evaluation of polymerase chain reaction-Restriction fragment length polymorphism for speciation and antifungal drug resistance. J Acad Clin Microbiologists. 2024;19(2):86–92.

[12] Saloojee H, Violari A. Clinical review Regular review HIV infection in children. BMJ [Internet]. 2001;323:670–4. Available from: www.unaids.org/wac/2000/wad00/files/wad2000Master/index.htm 10.1136/bmj.323.7314.670 PMC1121236 11566832

[13] Kidd SE, Abdolrasouli A, Hagen F. Fungal nomenclature: managing change is the name of the game. Open Forum Infect Dis. 2023;10(1). 10.1093/ofid/ofac559 PMC9825814 36632423

[14] Cuenca-Estrella M, Gomez-Lopez A, Alastruey-Izquierdo A, Bernal-Martinez L, Cuesta I, Buitrago MJ, et al. Comparison of the Vitek 2 antifungal susceptibility system with the clinical and laboratory standards Institute (CLSI) and European committee on antimicrobial susceptibility testing (EUCAST) broth microdilution reference methods and with the sensititre YeastOne and etest techniques for in vitro detection of antifungal resistance in yeast isolates. J Clin Microbiol. 2010;48(5):1782–6. 20220169 10.1128/JCM.02316-09 PMC2863906

[15] Nadagir SD, Chunchanur SK, Halesh LH, Yasmeen K, Chandrasekhar MR, Patil BS. Significance of isolation and drug susceptibility testing of non-Candida albicans species causing oropharyngeal candidiasis in HIV patients. Southeast Asian J Trop Med Public Health. 2008;39(3):492–5. 18564689

[16] Fidel PL. Candida-host interactions in HIV disease: implications for oropharyngeal candidiasis. Adv Dent Res. 2011;23(1):45–9. 21441480 10.1177/0022034511399284 PMC3144040

[17] Kukoyi O, Renner L, Powell J, Barry O, Prin M, Kusah J et al. Viral load monitoring and antiretroviral treatment outcomes in a pediatric HIV cohort in Ghana. BMC Infect Dis. 2016;16(1). 10.1186/s12879-016-1402-9 PMC4738803 26843068

[18] Leigh JE, Barousse M, Swoboda RK, Myers T, Hager S, Wolf NA et al. Candida-Specific Systemic Cell-Mediated Immune Reactivities in Human Immunodeficiency Virus-Positive Persons with Mucosal Candidiasis. J Infect Dis [Internet]. 2001;183(2):277–85. Available from: https://academic.oup.com/jid/article/183/2/277/848500 10.1086/317944 11120933

[19] Contaldo M, Romano A, Mascitti M, Fiori F, Della Vella F, Serpico R, et al. Association between denture stomatitis, Candida species and diabetic status. J Biol Regul Homeost Agents. 2019;33(3 Suppl 1):35–41. 31538448

[20] de Arruda Caceres N, Mazorra Coelho Vieira M, Fiorese Vieira I, Figueiredo Monteleone V, Jorge Moreira Neto L, Bonafe S et al. Opportunistic Infections in Aids Patients [Internet]. Available from: http://wwwimedpub.com

[21] McNulty KM, Plianrungsi J, Leigh JE, Mercante D, Fidel PL. Characterization of CD8 + T cells and microenvironment in oral lesions of human immunodeficiency virus-infected persons with oropharyngeal candidiasis. Infect Immun. 2005;73(6):3659–67. 15908395 10.1128/IAI.73.6.3659-3667.2005 PMC1111879

[22] Goomer RS, Maris T, Amiel D. Age-Related changes in the expression of Cadherin-11, the mesenchyme specific Calcium-Dependent cell adhesion molecule. Calcif Tissue Int. 1998;62:532–7. 9576982 10.1007/s002239900474

[23] Paoletti I, Fusco A, Grimaldi E, Perillo L, Coretti L, Di Domenico M, et al. Assessment of host defence mechanisms induced by Candida species. Int J Immunopathol Pharmacol. 2013;26(3):663–72. 24067462 10.1177/039463201302600309

[24] Menezes R, de Borges P, de Araujo AS, dos Pedroso LB. Röder DVD de B. Fatores relacionados a colonização Da Cavidade bucal de indivíduos Portadores do HIV Por espécies de Candida. Rev Inst Med Trop Sao Paulo. 2015;57(5):413–9. 26603229 10.1590/S0036-46652015000500008 PMC4660451

[25] Khedri S, Santos ALS, Roudbary M, Hadighi R, Falahati M, Farahyar S, et al. Iranian HIV/AIDS patients with oropharyngeal candidiasis: identification, prevalence and antifungal susceptibility of Candida species. Lett Appl Microbiol. 2018;67(4):392–9. 30019443 10.1111/lam.13052

[26] Ambe NF, Longdoh NA, Tebid P, Bobga TP, Nkfusai CN, Ngwa SB, et al. The prevalence, risk factors and antifungal sensitivity pattern of oral candidiasis in Hiv/aids patients in Kumba district hospital, South West region, Cameroon. Pan Afr Med J. 2020;36:1–14. 32774600 10.11604/pamj.2020.36.23.18202 PMC7392032

[27] Kothavade RJ, Kura MM, Valand AG, Panthaki MH. Candida tropicalis: its prevalence, pathogenicity and increasing resistance to fluconazole. J Med Microbiol. 2010;59:873–80. 20413622 10.1099/jmm.0.013227-0

[28] S KS, Natarajan S, Boaz K, Ramapuram J, Baliga S, Manaktala N et al. Candida profile in HIV-Positive children needs a dynamic clinical appraisal: A microbiological study. Res J Pharm Technol. 2023;16(1):423–8. 10.52711/0974-360X.2023.00072

[29] Mathavi S, Sasikala G, Kavitha A, Priyadarsini RI. CHROMagar as a primary isolation medium for rapid identification of Candida and its role in mixed Candida infection in sputum samples. Indian J Microbiol Res. 2016;3(2):141.

[30] Eraso E, Sahand IH, Villar-Vidal M, Marcos C, Dolores Moragues M, Madariaga L, et al. Usefulness of Candida ID2 agar for the presumptive identification of Candida Dubliniensis. Med Mycol. 2006;44(7):611–5. 17071554 10.1080/13693780600830691

